# Impacts of *LOC105371267* Variants on Breast Cancer Susceptibility in Northern Chinese Han Females: A Population-Based Case-Control Study

**DOI:** 10.1155/2021/4990695

**Published:** 2021-08-28

**Authors:** Linna Peng, Congmei Huang, Shishi Xing, Dandan Li, Chunjuan He, Yongjun He, Wei Yang, Tianbo Jin, Li Wang

**Affiliations:** ^1^Key Laboratory of Molecular Mechanism and Intervention Research for Plateau Diseases of Tibet Autonomous Region, School of Medicine, Xizang Minzu University, Xianyang, Shaanxi 712082, China; ^2^Department of Gynecology, The Second Affiliated Hospital of Hainan Medical University, No. 3, College Road, Longhua District, Haikou, Hainan, China; ^3^Department of Clinical Laboratory, The Affiliated Hospital of Xizang Minzu University, Xianyang, China

## Abstract

**Background:**

*LOC105371267*, also known as *PR-lncRNA1,* was reported to be a *p53*-regulated long noncoding RNA (lncRNA), which played an essential role in the pathogenesis of breast cancer (BC). We aimed to observe the potential association between *LOC105371267* polymorphisms and BC risk in Northern Chinese Han females.

**Methods:**

Totally, 555 healthy individuals and 561 patients with BC were recruited. Five candidate SNPs (rs6499221, rs3931698, rs8044565, rs3852740, and rs111577197) of *LOC105371267* were genotyped with the Agena MassARRAY system. Odds ratio (OR) and 95% confidence intervals (CIs) were applied to evaluate the relationship of *LOC105371267* genetic polymorphisms with BC susceptibility. Additionally, stratification analysis based on clinical features and haplotype analysis were also conducted. Finally, multifactor dimensionality reduction (MDR) analysis was performed to assess the SNP-SNP interaction among LOC105371267 variants, and false-positive report probability (FPRP) analysis was used to validate the result of this study.

**Results:**

In this study, rs3931698 was a protective factor of BC in total (GG homozygote: OR = 0.30, 95% CI: 0.11–0.82, *p*=0.018; recessive model: OR = 0.30, 95% CI: 0.11–0.84, *p*=0.021). In stratification analysis based on the average age of 52 years and clinical characteristics (PR status, III-IV TNM stage), rs3931698 was also demonstrated to be associated with BC susceptibility. In addition, rs6499221 and rs3852740 were also associated with BC susceptibility among patients at age <52 years and patients with BC in a positive status. Thus, the haplotype analysis had a negative result for the incidence of BC (*p* > 0.05), and haplotype consisting of rs8044565 and rs111577197 was nonsignificantly associated with the BC risk. Finally, MDR and FPRP analyses also validated the result of this study.

**Conclusion:**

Polymorphisms rs3931698, rs6499221, and rs3852740 of *LOC105371267* were found to be associated with the risk of BC in total, and stratification analysis in the Northern Chinese Han females suggested that *LOC105371267* variants might be helpful to predict BC progression.

## 1. Introduction

Breast cancer (BC), an important cause of human suffering and premature mortality among women, has been considered as one of the most prevailing cancers. At abroad, 1.7 million new cases have been diagnosed and 1.2 million people died of the disease per year [1–3]. At home, the health burden of cancer is increasing inescapably [4]. Due to its heterogeneous presentation, BC is classified into distinct subtypes, which differ in their unique biology, survival outcome, and related risk factors [5, 6]. The pathogenesis and overall prognosis of BC are multifactorial and result from interaction between modifiable factors such as breastfeeding and oral contraceptive use and nonmodifiable factors such as age, early menarche, late menopause, ethnicity, and genetic aberrations [7, 8]. Among the above-mentioned multiple factors, genetic factors were the major drivers in the genesis of BC [9–11]. *BRCA1* and *BRCA2* have proved to be the two classical susceptible genes in the BC hereditary [12, 13]. In addition, progesterone receptor (PGR) gene variants, MIR-375 gene, and NF-kB genetic variants were also demonstrated to be associated with BC risk [14–16]. Encouragingly, increasing attention has been concentrated on the role and susceptibility of lncRNAs in the BC pathogenesis [17–20]. For example, Ma et al. evaluated the association between BC risk and LncRNA *LINC01585* using a genome-wide association study (GWAS) method, and they suggested that the lncRNA probably served as a novel therapeutic target for BC [21]. Moreover, Peng et al. pointed out that lncRNA *MALAT1* polymorphisms were correlated with the risk of BC based on the association analysis in Chinese Han females [22], which indicated the crucial role of lncRNAs in BC pathogenesis. Among numerous kinds of lncRNAs, *LOC105371267*, a *p53*-regulated lncRNA [23, 24], whose RefSeq DNA sequence is NC_000016.10, was reported to be a probable new candidate susceptibility gene of BC in European women in a previous transcriptome-wide association study. However, there were no other studies on this gene, including genetic polymorphism of this gene. Thus, relative genetic roles of this gene are worth digging out. In the present study, we have a strong desire to explore the impact of *LOC105371267* genetic polymorphism on the risk of BC in Northern Chinese Han females conducting a case-control study. In addition, we also investigated the association between *LOC105371267* genetic polymorphisms and clinical characteristics of BC in stratification analysis. Finally, false-positive report probability (FPRP) analysis was conducted to validate the positive result in this study.

## 2. Patients and Methods

### 2.1. Study Population

In this case-control study, blood samples were collected from 561 female patients with BC and 555 female healthy individuals, who were consecutively recruited from the Shaanxi Provincial Cancer Hospital. All the BC cases were newly diagnosed as breast carcinoma through the histopathological examination, and none of them had undergone chemotherapy or radiotherapy before gathering samples. Moreover, those who had a history of other cancer or suffered from immunological, cardiovascular, or hematologic disorders were excluded. The control subjects were received from the physical examination center in the same hospital; they had no medical illness or family history of BC and were genetically unrelated to the included patients with BC. In addition, the demographic data of participants and the clinical information of patients with BC were acquired based on a standard questionnaire of clinical information, including age, estrogen receptor (ER), progesterone receptor (PR), Ki-67 status, tumor status, location and stage, lymph nodes metastasis, and distance metastasis. All participants signed informed consent, and this work was approved by the Ethics Committee of Xizang Minzu University. All experiments were conducted in accordance with the World Medical Association Declaration of Helsinki.

### 2.2. Gene Selection and SNPs Genotyping Assay

Total DNA isolation was undertaken from 5 mL of ethylenediamine tetraacetic acid (EDTA)-anticoagulated peripheral blood using the GoldMag whole blood genomic DNA purification kit (GoldMag Co. Ltd., Xi'an, China) according to the protocol of manufacturer and subsequently was stored at −80°C for the following analysis. We selected *LOC105371267*, SNPs referring to the genes, and SNPs reported in BC transcriptome analysis literature [24], by which our five candidate SNPs of *LOC105371267*, namely, rs6499221, rs3931698, rs8044565, rs3852740, and rs111577197, were near the SNPs demonstrated. Then, we identified the above five SNPs in 1000 Genomes Project database (https://www.ncbi.nlm.nih.gov/variation/tools/1000genomes/) based on CHB (Chinese Han in Beijing) data with minor allele frequency (MAF) > 0.05 and call rate >95% [25] in order to ensure the successful genotyping and valid statistical analysis in Northern Chinese Han females. Moreover, functional prediction analysis of these SNPs was performed using the web-based HaploReg v4.1 software (https://pubs.broadinstitute.org/mammals/haploreg/haploreg.php). Thus, we selected these five SNPs for subsequent genotyping. In this study, the genotyping was carried out with the Agena MassARRAY system (Agena, San Diego, CA, USA) as described in previous research [26] by two independent investigators. In addition, 10% of samples were randomly selected as blinded duplication to evaluate the accuracy of SNPs genotyping and exhibited 100% concordance. The used primers are summarized in Table S9.

### 2.3. Statistical Analyses

The differences in demographic and clinical data between cases and controls were assessed by Pearson's *χ*^2^ test and Student's *t*-test. Hardy–Weinberg equilibrium (HWE) analysis for each SNP among controls was conducted using Fisher's exact test for further analysis. Pearson's *χ*^2^ test was also used to analyze the difference in the allele and genotype frequencies for each polymorphism between patients with BC and healthy subjects. Accordingly, odds ratios (ORs) and 95% confidence intervals (CIs) were calculated using logistic regression analysis after the adjustment for age to evaluate the correlation between *LOC105371267* polymorphisms and BC risk using the PLINK v1.07 software. The Stata (version 11) software was used for the forest plot making in order to partly show the result of regression analysis intuitively. Meanwhile, multiple genetic models (genotype, dominant model, recessive model, and additive model) were utilized to estimate the relationship of *LOC105371267* SNPs with the susceptibility to BC. Moreover, we performed multiple stratified analyses in terms of average age at 52 years of the study population in this study (Table 1), ER (positive vs. negative), PR (positive vs. negative), lymph nodes metastasis (positive vs. negative), TNM stage [III-IV (*n* = 161) vs. I-II ], Ki-67 status (high vs. low), and tumor size (>2 cm vs. ≤ 2 cm). Additionally, the pairwise linkage disequilibrium (LD) was measured by the LD coefficient D′ using the Haploview v4.2 software. Haplotype analysis was conducted by logistic regression analysis using the PLINK v1.07 software. All statistical analyses were carried out using SPSS v 18.0 software (Armonk, New York City, NY, USA). Besides, *p* values adjusted for the false discovery rate (FDR) were also calculated in this study. Additionally, the noteworthy associations of the significant findings were evaluated using the FPRP evaluation method developed by Wacholder et al. [27] [FPRP cutoff value = 0.2, power OR = 2, and prior probability levels = (0.25, 0.1, 0.01, 0.001, 0.0001)]. Finally, the MDR analysis was carried out using MDR software (version 3.0.2) to evaluate the SNP-SNP interactions among these three candidate SNPs. All statistical tests were two-sided, and *p* < 0.05 was considered statistically significant. The *p* value analyzed by the MDR analysis was calculated using *χ*^2^ test. Flow diagram of study design is shown in Figure S1.

## 3. Results

### 3.1. Characteristics of Study Population and SNP Identification

A total of 1116 female participants (561 patients with BC and 555 controls) were recruited in the current study. The baseline characteristics of these subjects are exhibited in Table 1. We noted that no significant difference was detected between BC cases and controls (*p* < 0.05) in terms of age. Among 561 BC cases with available ER, PR, Ki-67, tumor size, tumor location, lymph nodes metastasis, distance metastasis, TNM stage, and primary or recurrent information, 380 (67.7%) cases were ER positive, 328 (58.5%) cases were PR positive, 371 (66.1%) cases had high Ki-67, 238 (42.4%) cases had tumor size > 2 cm, 267 (47.6%) cases were lymph nodes metastasis positive, 517 (92.2%) cases had distance metastasis of M0, 366 (65.2%) cases were at I-II TNM stage, and 424 (75.6%) cases were primary. Five SNPs of *LOC105371267* (rs6499221, rs3931698, rs8044565, rs3852740, and rs111577197) were screened according to the criteria described above and successfully genotyped in included samples. The fundamental information of these SNPs is displayed in Table 2, and the genotypes frequency of all SNPs in control group conforms to HWE (*p* < 0.05), which then could be used as a basis for further study. Moreover, there were no significant differences in allele frequencies between patients and healthy controls (*p* > 0.05) with or without the FDR test, implying that these SNPs were not susceptible to BC in the allele model.

### 3.2. Associations between *LOC105371267* Polymorphisms and BC Risk

**Figure 1:**
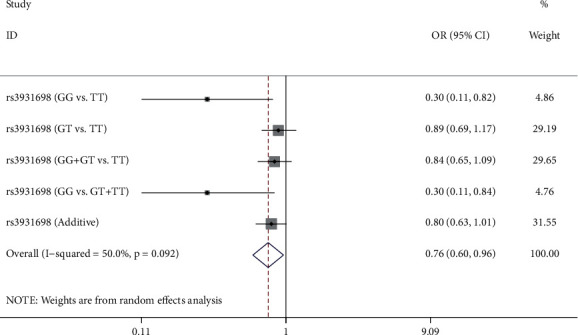
Association analysis results of genetic polymorphisms of *LOC105371267* rs3931698 and breast cancer susceptibility.

The logistic regression model was used to evaluate the associations between *LOC105371267* SNPs and the risk of BC based on the adjustment for age. As can be seen from Table S1 and Figure 1, the homozygote of rs3931698 (GG vs. TT) had a 0.3-fold decreased BC risk (OR = 0.30, 95% CI: 0.11–0.82, *p*=0.018). Similarly, a 0.3-fold reduced risk was also observed for rs3931698 in the recessive model (OR = 0.30, 95% CI: 0.11–0.84, *p*=0.021), yet FDR test did not prove this positive result. Negatively, there was no dramatically statistical difference between BC risk and remaining SNPs (rs6499221, rs8044565, rs3852740, and rs111577197) in any genetic model (*p* > 0.05) with or without the FDR test (*p* > 0.05).

### 3.3. Stratification Analyses

**Figure 2:**
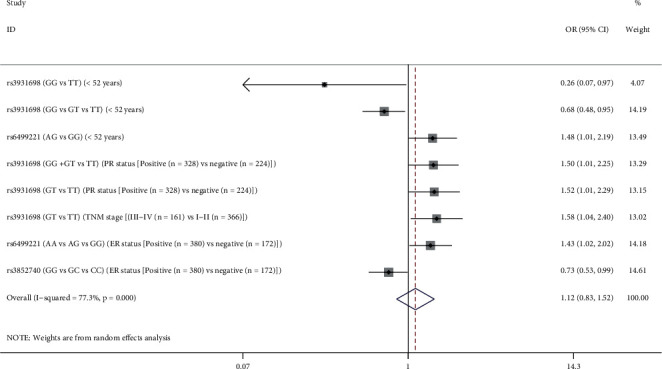
Positive results of stratification analysis between *LOC105371267* polymorphisms and breast cancer risk based on age of 52 years, PR status, TNM stage, and ER status.

According to the epidemiology report of BC, the mean age at diagnosis of breast cancer in China is approximately within 45–55 years [4]; besides, combined with the average age of the study subjects enrolled in this study, the subsequent stratified analyses based on age of 52 years were performed. As shown in Table S2 and Figure 2, rs3931698 exhibited significant association with a decreased BC risk among patients at age <52 years in homozygous genotype (GG vs. TT, OR = 0.26, 95% CI: 0.71–0.97, *p*=0.045) and additive model (OR = 0.68, 95% CI: 0.48–0.95, *p* > 0.025) with adjustments for age. We also found that heterozygous genotype of rs6499221 was associated with an increased BC risk at BC cases at age <52 years (AG vs. GG, OR = 1.48, 95% CI: 1.01–2.09, *p*=0.046); however, we got a negative result after the FDR test (*p* > 0.05). Similarly, the above two SNPs had a negative result on the BC susceptibility analysis for patients with BC at age ≥52 years. As for the other three SNPs (rs8044565, rs3852740, and rs111577197), no significant association was detected between age and susceptibility to BC with or without the FDR test (*p* > 0.05), either.

Additionally, the correlation between clinical characteristics and BC susceptibility was also assessed. As shown in Table S3 and Figure 2, the heterozygous genotype and dominant model of rs3931698 were correlated with an increased possibility of PR-positive BC (GT vs. TT : OR = 1.52, 95% CI: 1.01–2.29, *p*=0.043), and likewise rs6499221 increased the BC feasibility of ER-positive patients in the additive genetic model (OR = 1.43, 95% CI: 1.02–2.02, *p*=0.041). However, the additive model of rs3582740 was associated with a decreased probability of ER-positive BC (OR = 0.73, 95% CI: 0.53–0.99, *p*=0.043), which indicated the poor effect of rs3582740 on ER-positive BC. On the other hand, there was no correlation between the other two candidate SNPs (rs8044565 and rs111577197) of *LOC105371267* and ER or PR status (Tables S4–S8), and no positive result existed after the FDR test (*p* > 0.05) in this stratified analysis based on the PR or ER status of BC.

Afterwards, we further evaluated the impacts of *LOC105371267* SNPs on the severity of BC according to TNM stage (III-IV/I-II). As shown in Table S3 and Figure 2, the results revealed that heterozygous genotype of rs3931698 was overrepresented in patients with clinical III-IV stage compared to those with I-II stage (OR = 1.58, 95% CI: 1.04–2.40, *p*=0.033), which indicated the poor effect of rs3931698 on TNM stage of BC. Nevertheless, after the FDR test, the positive result no longer existed. Negatively, there was no correlation between other SNPs and TNM stage of BC (Tables S5–S8). Additionally, no statistical difference was estimated between the selected five SNPs in *LOC105371267* and tumor size, Ki-67 status, or lymph nodes metastasis based on the stratification analyses (Tables S4–S8) with or without the FDR test.

### 3.4. Haplotype Analysis of *LOC105371267* Polymorphisms

**Figure 3:**
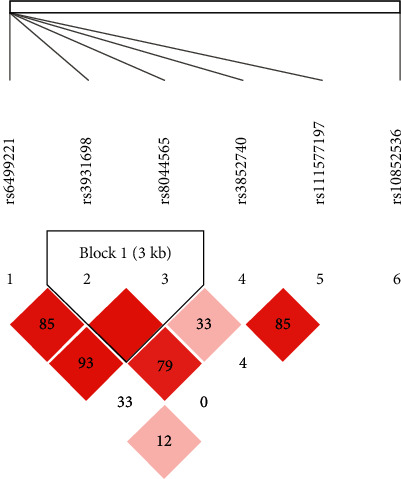
The LD status (D′) of two SNPs of *LOC105371267*. The number in the diamonds is the LOD score of D′. The LD value is determined by D′ > 0.8 analyzed by Haploview software 4.2. LD haplotype analysis provides the basis for the selection of sites for association analysis.

The LD and corresponding haplotypes analysis were further investigated by Haploview software to explore the combined effect of these five SNPs in *LOC105371267* on BC risk. Our findings implied that only two SNPs, rs3931698 and rs8044565, were in high LD and formed three haplotypes (TC, GT, and TT) (Figure 3). However, none of haplotypes was related to the incidence of BC in the condition with or without the FDR test (*p* > 0.05, Table S10).

### 3.5. FPRP Analysis

As recommended by Wacholder et al. [27], only FPRP value is less than the preset threshold (0.2), which means that the false-positive rate of the positive result is lower than the expected value, and the positive result is noteworthy. Thus, we set 0.2 as an FPRP threshold. Besides, according to the FPRP analysis result in this study, we assigned a prior probability of 0.1 to detect OR of 2 for an association between BC risk and genotypes under investigation, although the setting of OR value is more conservative than that suggested by Wacholder et al. (OR = 1.5) [27]. As can be seen from Table 3, when OR value was 2, the effect of additive model of rs3931698 on BC risk under the subgroup (patients at age <52 years) conformed to the notable association, whose FPRP level was <0.2 under the prior probability level of 0.1; furthermore, other positive results still had notable associations since the FPRP value was <0.2 although the prior probability level was 0.25.

### 3.6. MDR Analysis

**Figure 4:**
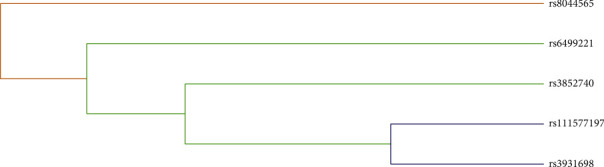
The dendrogram of the SNP-SNP interaction of five SNPs on *LOC105371267* gene. The bluer the string color, the more redundant the effect between those five SNPs. Contrarily, the redder the color, the more the synergy effect between those five SNPs.

Finally, we conducted the MDR analysis to explore the SNP-SNP interaction among five loci (rs111577197, rs3852740, rs3931698, rs6499221, rs8044565) in the *LOC105371267* gene to better evaluate the effect of *LOC105371267* variants on BC risk. As shown in Table 4 and Table S11, the larger the “CV consistency” value and “1/0 ratio” value, the stronger the interaction among these SNPs. A model consisting of five loci (rs111577197, rs3852740, rs3931698, rs6499221, rs8044565) with the largest “CV consistency” value (10/10) could be the best multilocus model, and the best genotype combination of this model was rs111577197-TT, rs3852740-CC, rs3931698-GT, rs6499221-AG, rs8044565-TC. At the same time, the impact of this best model on the risk of BC prediction was significant (*p* < 0.0001). Likewise, as shown in Figure 4, the bluer the string color, the greater the redundancy effect among those five SNPs. Contrarily, the redder the color, the greater the synergy effect among those five SNPs. Furthermore, we could observe that a strong redundancy effect existed between rs3931698 and rs111577197. The redundancy effect among other loci decreased gradually, and the synergy effect increased gradually.

## 4. Discussion

BC is a serious threat to women's health. Genetic factors played an important role in the etiology of BC. Fortunately, numerous researchers have concentrated on elucidating the correlations between lncRNAs and susceptibility to BC in recent years. For example, Li et al. conducted a GWAS-based association analysis and concluded that polymorphism rs12325489-C.T in the LncRNA ENST00000515084 Exon was found to modulate BC risk in populations including the Northern Chinese population [28]. Besides, Zheng et al. reported that LncRNA *MEG3* rs3087918 was associated with a decreased BC risk in the Chinese population [29]. Liu et al. concluded that LncRNA H19 variants were associated with the BC risk by a meta-analysis [30].

In this study, we analyzed the relationship between lncRNA *LOC105371267*, with the accession number of NC_000016, and BC susceptibility among the Northern Chinese Han females. To the best of our knowledge, this is the first study to explore the association between *LOC105371267* SNPs (rs6499221, rs3931698, rs8044565, rs3852740, and rs111577197) and BC risk. In this study, five candidate SNPs (rs6499221, rs3931698, rs8044565, rs3852740, and rs111577197) were successfully genotyped. We found that carriers with rs3931698-G allele might have a decreased incidence of BC in total. Stratified by age, rs6499221 was associated with an increased BC risk while rs3931698 was associated with a decreased BC risk among patients with BC at age <52 years. Meanwhile, an increased risk was also observed between rs3931698 and other PR-positive and III-IV stage BC. Polymorphisms of rs6499221 and rs3852740 played protective and dangerous roles in the additive model in ER-positive patients, respectively. Besides, the FDR test and FPRP analysis were also conducted to validate our result. Unfortunately, all the results of FDR test were negative. Encouragingly, certain FPRP analysis result could validate our result, which can be seen in the Results. Likewise, the MDR analysis also concluded that the model (rs111577197, rs3852740, rs3931698, rs6499221, rs8044565) with the largest “CV consistency” value (10/10) could be the best multilocus model, which showed a significant association with BC risk. Therefore, SNPs (rs3931698, rs6499221, and rs3852740) of *LOC105371267* might be associated with the occurrence and development of BC. However, no significant relationship was found for rs111577197 and rs8044565 of *LOC105371267* with BC risk in this study.

Notably, *LOC105371267* is a *p53*-regulated lncRNA. It has been much demonstrated that tumor suppressor *p53* played an essential role in molecular mechanisms of cancer progression [31–33]. Noteworthily, *p53*-regulated lncRNAs were reported to contribute to the occurrence of different types of cancers [34]. For example, Liu et al. highlighted the fact that LncRNA *LOC285194*, a p53-regulated lncRNA, served as a tumor suppressor in colon cancer via mediating the expression of miR-211 [35].

Most importantly, Sánchez et al. highlighted the fact that *LOC105371267* could enhance cell apoptosis and cell cycle arrest by promoting the *p53* signaling activation. Specifically, they argued that *PR-lncRNA1* regulated the *p53* transcriptional network by the efficient binding of *p53* to some of its target genes [23]. Furthermore, Li and Richard previously also pinpointed that *PR-lncRNA1* interacted with a sequence-specific RNA binding protein, Sam68, and this complex could promote the *p53*-mediated transcription in human colon carcinoma cell lines [36]. These lines of evidence suggest that *LOC105371267* could be of pathogenic importance in the occurrence and development of BC. For the susceptibility of *LOC105371267* SNPs to cancer or noncancer diseases, there was no other specific research on it so far, but a transcriptome-wide association study is available [24]. According to the retrieval of the potential function of these five SNPs (rs6499221, rs3931698, rs8044565, rs3852740, and rs111577197) on *LOC105371267* gene in the dbSNP database (https://www.ncbi.nlm.nih.gov/snp/), all of the candidate SNPs in this study are located in the intron sequence. Several studies have proved that the intronic SNPs conferred susceptibilities by affecting gene expression [37], and the expression and function of lncRNA were affected by SNPs [38], so these SNPs might have potential function in *LOC105371267* expression level. Nevertheless, the detailed roles of *LOC105371267* or SNPs (rs6499221, rs3931698, rs8044565, rs3852740, and rs111577197) in BC risk remain to be explored in further study.

There is no doubt that age factor has been identified as a prominent risk factor in the BC initiation [39]. Recently, a significant increase in BC rates has been observed among premenopausal subjects [40]. There were also some studies showing that patients with BC at the oldest age were more vulnerable to rapid deterioration [41]. For example, Unlu et al. pointed out that older women tended to have a higher BC risk compared with younger ones [42]. However, in this study, the findings that *LOC105371267* SNPs rs3931698 and rs6499221 were related to the decreased and increased risk of BC, respectively, among patients at age <52 years indicated that women at age <52 years with rs3931698 and rs6499221 variants were more susceptible to BC. Importantly, the ER and PR were the decisive therapeutic targets in the treatment of BC. Additionally, Ki-67, lymph nodes metastasis, TNM stage, and tumor size were also linked with the BC pathogenesis [43–45]. Moreover, we also found that *LOC105371267* SNPs (rs3931698, rs6499221, and rs3852740) of *LOC105371267* might be associated with ER status, PR status, and TNM stage of BC in this study, which can be seen in the Results.

Although several positive associations were observed in this study, some limitations still should be considered. First of all, since all participants were enrolled in the same hospital and were Northern Chinese females, the inherent selection bias cannot be excluded and our results cannot permit extrapolation of the results to other ethnic groups. Then, the comprehensive clinical information and environmental factors should be included. Next, the precise molecular mechanisms of *LOC105371267* polymorphisms in BC progression remain to be deciphered. Last but not least, due to the limited sample size, the statistical effect is not enough, and thus larger sample size and in vitro functional experiment are needed to evaluate the association of *LOC105371267* polymorphisms and BC susceptibility in Northern Chinese Han females. Despite the limitations mentioned above, our study was only a preliminary study which shed light on the relationship between *LOC105371267* polymorphisms and BC risk. The results of our study might provide a foundation for future studies on the relationship of *LOC105371267* polymorphisms with BC pathogenesis.

## 5. Conclusion

In summary, this study first shed light on the impact of polymorphisms rs3931698, rs6499221, and rs3852740 of *LOC105371267* on BC susceptibility in Northern Chinese Han females, suggesting that *LOC105371267* variants might be genetic markers of BC risk, which is benefit for the diagnosis and prognosis of BC.

## Figures and Tables

**Table 1:** Characteristics of breast cancer cases and cancer-free controls.

Variables	Breast cancer patients (*n* = 561)	Control (*n* = 555)	*p* ^a^
Age (years) (mean ± SD)	52.04 ± 9.82	51.84 ± 9.76	0.738
*ER status, n (%)*			
Positive	380 (67.7)		
Negative	172 (30.7)		
Unavailable	9 (1.6)		

*PR status, n (%)*			
Positive	328 (58.5)		
Negative	224 (39.9)		
Unavailable	9 (1.6)		

*Ki67 status, n (%)*			
High	371 (66.1)		
Low	154 (27.5)		
Unavailable	36 (6.4)		

*Tumor size (cm), n (%)*			
>2	238 (42.4)		
≤2	206 (36.7)		
Unavailable	117 (20.9)		

*Tumor location, n (%)*			
Right	267 (47.6)		
Left	284 (50.6)		
Bilateral	8 (1.4)		
Unavailable	2 (0.4)		

*Lymph nodes metastasis, n (%)*			
Positive	277 (49.4)		
Negative	279 (49.7)		
Unavailable	5 (0.9)		

*Distance metastasis, n (%)*			
M0	517 (92.2)		
M1	39 (7.0)		
Unavailable	5 (0.9)		

*TNM stage, n (%)*			
I-II	366 (65.2)		
III-IV	161 (28.7)		
Unavailable	34 (6.1)		

*Primary or recurrent, n (%)*			
Primary	424 (75.6)		
Recurrent	22 (3.9)		
Unavailable	112 (20.5)		

ER: estrogen receptor; RP: progesterone receptor; SD: standard deviation. Note: ^a^*p* values were calculated by independent samples *t*-test; empty cells indicate data loss.

**Table 2:** Basic characteristics about *LOC105371267* candidate SNPs and relationship with risk of breast cancer in allele model.

SNPs	Chromosome	Position	Type	Allele (minor/major)	MAF (case/control)	HWE-*p*^a^	OR (95% CI)	*p* ^b^	FDR test	HaploReg
rs6499221	16q12.2	53036124	Intron	A/G	0.19/0.19	0.49	1.02 (0.83–1.26)	0.872	1.453	Enhancer histone marks, motifs changed

rs3931698	16q12.2	53036913	Intron	G/T	0.14/0.16	0.64	0.80 (0.64–1.02)	0.074	0.370	Enhancer histone marks, DNase, motifs changed

rs8044565	16q12.2	53040078	Intron	C/T	0.25/0.24	1.00	1.01 (0.83–1.22)	0.961	0.961	Motifs changed

rs3852740	16q12.2	53044259	Intron	G/C	0.20/0.20	0.69	0.98 (0.80–1.21)	0.874	1.093	Promoter histone marks, enhancer histone marks, DNase, proteins bound, motifs changed

rs111577197	16q12.2	53049243	Intron	T/C	0.22/0.21	0.80	1.09 (0.89–1.33)	0.433	1.083	Enhancer histone marks, motifs changed

SNP: single-nucleotide polymorphism; MAF: minor allele frequency; HWE: Hardy–Weinberg equilibrium; OR: odds ratio; 95% CI: 95% confidence interval. Note: OR and 95% CI were computed by logistic regression analysis with adjustments for age. ^a^*p* values for the Hardy–Weinberger equilibrium (HWE) test, calculated by Fisher's exact test. ^b^*p* values were calculated by two-sided *χ*2 test after adjustment for age with logistic regression analysis.

**Table 3:** FPRP evaluation for association between *LOC105371267* variants and breast cancer risk.

Group (subgroup)/variants/genotype	OR (95% CI)	Power^a^, OR = 2	OR = 2				
			Prior probability level				
			0.25	0.1	0.01	0.001	0.0001
Overall/rs3931698/GT vs. TT	0.30 (0.11–0.82)	0.160	0.284	0.544	0.929	0.993	0.999
Overall/rs3931698/GG vs. GT + TT	0.30 (0.11–0.84)	0.165	0.262	0.516	0.922	0.992	0.999
Age <52 years/rs3931698/GG vs. TT	0.26 (0.17–0.97)	0.165	0.449	0.71	0.964	0.996	0.999
Age <52 years/rs3931698/additive	0.68 (0.48–0.95)	0.964	**0.069**	**0.182**	0.709	0.961	0.996
Age <52 years/rs6499221/AG vs. GG	1.48 (1.01–2.19)	0.934	**0.138**	0.325	0.841	0.982	0.998
PR status/rs3931698/GT vs. TT	1.52 (1.01–2.29)	0.905	**0.13**	0.31	0.832	0.98	0.998
PR status/rs3931698/GG +GT vs. TT	1.50 (1.01–2.25)	0.918	**0.141**	0.329	0.844	0.982	0.998
TNM stage/rs3931698/GT vs. TT	1.58 (1.04–2.40)	0.865	**0.1**	0.25	0.785	0.974	0.997
ER status/rs6499221/additive	1.43 (1.02–2.02)	0.972	**0.116**	0.282	0.812	0.978	0.998
ER status/rs3852740/additive	0.73 (0.53–0.99)	0.993	**0.115**	0.28	0.811	0.977	0.998

OR: odd ratio; 95% CI: 95% confidence interval; ER: estrogen receptor; RP: progesterone receptor. Note: OR and 95% CI were computed by logistic regression analysis with adjustments for age. ^a^ statistical power to detect an OR of 2; FPRP value < 0.2 in bold.

**Table 4:** MDR analysis for impact of *LOC105371267* variants on risk of breast cancer.

Model	Training bal. acc.	Testing bal. acc.	CV consistency	Accuracy	Sensitivity	Specificity	OR (95% CI)	*p* ^a^
rs3931698	0.518	0.495	7/10	0.518	0.739	0.295	1.184 (0.911, 1.538)	0.206

rs3931698, rs6499221	0.531	0.481	6/10	0.529	0.476	0.583	1.269 (1.002, 1.607)	0.054

rs3852740, rs3931698, rs6499221	0.544	0.483	4/10	0.542	0.700	0.381	1.437 (1.121, 1.842)	**0.005**

rs111577197, rs3852740, rs3931698, rs8044565	0.559	0.480	7/10	0.556	0.629	0.482	1.576 (1.242, 2.001)	**0.0001**

rs111577197, rs3852740, rs3931698, rs6499221, rs8044565	0.574	0.497	10/10	0.570	0.602	0.538	1.761 (1.389, 2.232)	**<0.0001**

MDR: multifactor dimensionality reduction; SNP: single-nucleotide polymorphism; bal. acc.: balanced accuracy; CV: cross-validation; OR: odds ratio; CI: confidence interval. Note: ^a^interactions were validated by 1000 permutation tests. All *p values* in this study were two-tailed. Bold values mean statistical significance (*p* < 0.05).

## Data Availability

All the data regarding the findings are available within the manuscript. Anyone who is interested in the information should contact the corresponding author.

## Abbreviations

lncRNA: Long noncoding RNA
BC: Breast cancer
SNP: Single-nucleotide polymorphism
OR: Odds ratio
CI: Confidence interval
ER: Estrogen receptor
PR: Progesterone receptor
MAFs: Minor allele frequencies
HWE: Hardy-Weinberg equilibrium
LD: Linkage disequilibrium
SD: Standard deviation
FPRP: False-positive report probability
MDR: Multifactor dimensionality reduction
CV: Cross-validation.

## Supplementary Materials

Supplementary Materials Figure S1: flow diagram of study design. Table S1: association analysis results of genetic polymorphisms of *LOC105371267* and breast cancer susceptibility. Table S2: results of stratification analysis between *LOC105371267* polymorphisms and breast cancer risk by age of 52 years. Table S3: SNPs rs3931698 (T > G), rs6499221 (G > A), and rs3852740 (C > G) of *LOC105371267* were positively correlated with the PR status, TNM stage, and ER status of breast cancer. Table S4: relationships of *LOC105371267* rs3931698 (T > G) and clinical characteristics of breast cancer risk. Table S5: relationships of *LOC105371267* rs6499221 (G > A) and clinical characteristics of breast cancer. Table S6: relationships of *LOC105371267* rs8044565 (T > C) and clinical characteristics of breast cancer. Table S7: relationships of *LOC105371267* rs3852740 (C > G) and clinical characteristics of breast cancer. Table S8: relationships of *LOC105371267* rs111577197 and clinical characteristics of breast cancer. Table S9: primers used in this study. Table S10: results of association analysis of haplotype of *LOC105371267* and the risk of breast cancer. Table S11: the genotype combination of SNP-SNP interaction among variants of *LOC105371267* in breast cancer risk analysis using MDR method. .
