# Supplementary material for: Impacts of LOC105371267 Variants on Breast Cancer Susceptibility in Northern Chinese Han Females: A Population-Based Case-Control Study
Source: J Oncol. 2021 Aug 28;2021:4990695. doi: 10.1155/2021/4990695 (PMC8407995; doi:10.1155/2021/4990695)
Supplement: Supplementary Materials — Figure S1: flow diagram of study design. Table S1: association analysis results of genetic polymorphisms of LOC105371267 and breast cancer susceptibility. Table S2: results of stratification analysis between LOC105371267 polymorphisms and breast cancer risk by age of 52 years. Table S3: SNPs rs3931698 (T > G), rs6499221 (G > A), and rs3852740 (C > G) of LOC105371267 were positively correlated with the PR status, TNM stage, and ER status of breast cancer. Table S4: relationships of LOC105371267 rs3931698 (T > G) and clinical characteristics of breast cancer risk. Table S5: relationships of LOC105371267 rs6499221 (G > A) and clinical characteristics of breast cancer. Table S6: relationships of LOC105371267 rs8044565 (T > C) and clinical characteristics of breast cancer. Table S7: relationships of LOC105371267 rs3852740 (C > G) and clinical characteristics of breast cancer. Table S8: relationships of LOC105371267 rs111577197 and clinical characteristics of breast cancer. Table S9: primers used in this study. Table S10: results of association analysis of haplotype of LOC105371267 and the risk of breast cancer. Table S11: the genotype combination of SNP-SNP interaction among variants of LOC105371267 in breast cancer risk analysis using MDR method. . [file 4990695.f1.zip › 4990695.f1/Supplemental Tables...docx]

**Table S1.** Association analysis results of genetic polymorphisms of *LOC105371267* and breast cancer susceptibility.

| SNPs | Model | Genotype | BC patients  n (%) | Controls  n (%) | OR (95% CI) | *p*- value ^a^ | FDR test |
| --- | --- | --- | --- | --- | --- | --- | --- |
| rs3931698 | Genotypes | GG | 5 (0.9%) | 16 (2.9%) | 0.30 (0.11-0.82) | **0.018** | 0.090 |
|  |  | GT | 141 (25.1%) | 148 (26.7%) | 0.89 (0.69-1.17) | 0.430 | 0.430 |
|  |  | TT | 415 (74.0%) | 391 (70.5%) | 1 |  |  |
|  | Dominant | GG+GT | 146 (26.0%) | 164 (29.6%) | 0.84 (0.65-1.09) | 0.189 | 0.236 |
|  |  | TT | 415 (74.0%) | 391 (70.5%) | 1 |  |  |
|  | Recessive | GG | 5 (0.9%) | 16 (2.9%) | 0.30 (0.11-0.84) | **0.021** | 0.053 |
|  |  | GT+TT | 556 (99.1%) | 536 (97.2%) | 1 |  |  |
|  | Additive | - | - | - | 0.80 (0.63-1.01) | 0.064 | 0.107 |
| rs8044565 | Genotypes | CC | 38 (6.8%) | 33 (5.9%) | 1.13 (0.69-1.84) | 0.635 | 1.588 |
|  |  | CT | 199 (35.4%) | 205 (36.9%) | 0.95 (0.74-1.22) | 0.682 | 1.137 |
|  |  | TT | 324 (57.8%) | 317 (57.1%) | 1.00 |  |  |
|  | Dominant | CC+CT | 237 (42.2%) | 238 (42.8%) | 0.97 (0.77-1.24) | 0.827 | 1.034 |
|  |  | TT | 324 (57.8%) | 317 (57.1%) | 1.00 |  |  |
|  | Recessive | CC | 38 (6.8%) | 33 (5.9%) | 1.15 (0.71-1.86) | 0.571 | 2.855 |
|  |  | CT+TT | 523 (93.2%) | 522(94.0%) | 1.00 |  |  |
|  | Additive | - | - | - | 1.01 (0.83-1.22) | 0.961 | 0.961 |
| rs3852740 | Genotypes | GG | 23 (4.1%) | 20 (3.6%) | 1.12 (0.60-2.08) | 0.720 | 0.900 |
|  |  | GC | 175 (31.2%) | 182 (32.8%) | 0.94 (0.73-1.21) | 0.612 | 3.060 |
|  |  | CC | 363 (64.7%) | 353 (63.6%) | 1.00 |  |  |
|  | Dominant | GG+GC | 198 (35.3%) | 202 (36.4%) | 0.66 (0.75-0.22) | 0.709 | 1.182 |
|  |  | CC | 363 (64.7%) | 353 (63.6%) | 1.00 |  |  |
|  | Recessive | GG | 23 (4.1%) | 20 (3.6%) | 1.14 (0.62-2.11) | 0.666 | 1.665 |
|  |  | GC+CC | 538 (95.9%) | 535 (96.4%) | 1.00 |  |  |
|  | Additive | - | - | - | 0.98 (0.80-1.21) | 0.865 | 0.865 |
| rs111577197 | Genotypes | TT | 30 (5.3%) | 25 (4.5%) | 1.22 (0.71-2.13) | 0.472 | 1.180 |
|  |  | TC | 188 (33.5%) | 180 (32.4%) | 1.06 (0.83-1.37) | 0.630 | 0.630 |
|  |  | CC | 343 (61.1%) | 350 (63.1%) | 1.00 |  |  |
|  | Dominant | TT+TC | 218 (38.8%) | 205 (36.9%) | 1.08 (0.85-1.38) | 0.515 | 0.858 |
|  |  | CC | 343 (61.1%) | 350 (63.1%) | 1.00 |  |  |
|  | Recessive | TT | 30 (5.3%) | 25 (4.5%) | 1.20 (0.70-2.07) | 0.515 | 0.644 |
|  |  | TC+CC | 531 (94.6%) | 530 (95.5%) | 1.00 |  |  |
|  | Additive | - | - | - | 1.08 (0.89-1.32) | 0.435 | 2.175 |
| rs6499221 | Genotypes | AA | 18 (3.2%) | 23 (4.1%) | 0.79 (0.42-1.49) | 0.469 | 0.782 |
|  |  | AG | 182 (32.4%) | 166 (29.9%) | 1.11 (0.86-1.43) | 0.422 | 1.055 |
|  |  | GG | 361 (64.3%) | 366 (65.9%) | 1.00 |  |  |
|  | Dominant | AA+AG | 200 (36.0%) | 189 (34.0%) | 1.07 (0.84-1.37) | 0.582 | 0.728 |
|  |  | GG | 361 (64.3%) | 366 (65.9%) | 1.00 |  |  |
|  | Recessive | AA | 18 (3.2%) | 23 (4.1%) | 0.77 (0.41-1.43) | 0.403 | 2.015 |
|  |  | AG+GG | 543 (96.8%) | 532 (95.8%) | 1.00 |  |  |
|  | Additive | - | - | - | 1.02 (0.83-1.26) | 0.851 | 0.851 |

Abbreviations: SNP: single-nucleotide polymorphism; MAF: minor allele frequency; HWE: Hardy–Weinberg equilibrium; OR: odds ratio; 95% CI: 95% confidence interval; FDR: false discovery rate.

Note: OR and 95% CI were computed by logistic regression analysis with adjustments for age.

^a^ *p* values for the Hardy–Weinberger equilibrium (HWE) test, calculated by Fisher’s exact test.

^b^ *p* values were calculated by two -sided χ2 test after adjusted by age with logistic regression analysis.

Bold means statistical significance.

**Table S2.** Results of stratification analysis between *LOC105371267* polymorphisms and breast cancer risk by age of 52 years.

| SNPs | Model | Genotype | ≥ 52 years | | | | < 52 years | | | | | |
| --- | --- | --- | --- | --- | --- | --- | --- | --- | --- | --- | --- | --- |
|  |  |  | BC patients  (n = 295) | Controls  (n = 293) | OR (95% CI) | *p*-value a | FDR test | BC patients  (n = 266) | Controls  (n = 262) | OR (95% CI) | *p-*value a | FDR test |
| rs8044565 | Genotypes | CC | 18 (6.10%) | 17 (5.80%) | 0.95 (0.47-1.91) | 0.882 | 1.103 | 20 (7.52%) | 16 (6.11%) | 1.33 (0.67-2.67) | 0.418 | 0.523 |
|  |  | CT | 106 (35.93%) | 123 (41.98%) | 0.78 (0.55-1.09) | 0.141 | 0.705 | 93 (34.96%) | 82 (31.30%) | 1.22 (0.84-1.77) | 0.292 | 0.487 |
|  |  | TT | 171 (57.97%) | 153 (52.22%) | 1 |  |  | 153 (57.52%) | 164 (62.60%) | 1 |  |  |
|  | Dominant | CC+CT | 124 (42.03%) | 140 (47.78%) | 0.80 (0.57-1.10) | 0.170 | 0.425 | 113 (42.48%) | 98 (37.40%) | 1.24 (0.87-1.76) | 0.230 | 1.150 |
|  |  | TT | 171 (57.97%) | 153 (52.22%) | 1 |  |  | 153 (57.52%) | 164 (62.60%) | 1 |  |  |
|  | Recessive | CC | 18 (6.10%) | 17 (5.80%) | 1.05 (0.53-20.89) | 0.879 | 1.465 | 20 (7.52%) | 16 (6.11%) | 1.24 (0.63-2.50) | 0.533 | 0.533 |
|  |  | CT+TT | 277 (93.90%) | 276 (94.20%) | 1 |  |  | 246 (92.48%) | 246 (93.89%) | 1 |  |  |
|  | Additive | - | - | - | 0.87 (0.66-1.13) | 0.904 | 1.130 | - | - | 1.18 (0.90-1.56) | 0.230 | 0.575 |
| rs3852740 | Genotypes | GG | 14 (4.75%) | 11 (3.75%) | 1.26 (0.56-2.83) | 0.584 | 1.460 | 9 (3.38%) | 9 (3.44%) | 0.98 (0.34-2.54) | 0.965 | 1.206 |
|  |  | GC | 93 (31.53%) | 97 (33.11%) | 0.95 (0.67-1.35) | 0.775 | 1.292 | 82 (30.83%) | 85 (32.44%) | 0.93 (0.64-1.35) | 0.697 | 3.485 |
|  |  | CC | 188 (63.73%) | 185 (63.14%) | 1 |  |  | 175 (65.79%) | 168 (64.12%) | 1 |  |  |
|  | Dominant | GG+GC | 107 (36.27%) | 108 (36.86%) | 0.98 (0.70-1.38) | 0.914 | 0.914 | 91 (34.21%) | 94 (35.88%) | 0.93 (0.65-1.34) | 0.708 | 1.770 |
|  |  | CC | 188 (63.73%) | 185 (63.14%) | 1 |  |  | 175 (65.79%) | 168 (64.12%) | 1 |  |  |
|  | Recessive | GG | 14 (4.75%) | 11 (3.75%) | 0.28 (0.57-2.86) | 0.552 | 2.76 | 9 (3.38%) | 9 (3.44%) | 1.00 (0.39-2.58) | 0.995 | 0.995 |
|  |  | GC+CC | 281 (95.25%) | 282 (96.25%) | 1 |  |  | 257 (96.62%) | 253 (96.56%) | 1 |  |  |
|  | Additive | - | - | - | 1.02 (0.77-1.35) | 0.904 | 1.130 | - | - | 0.95 (0.70-1.30) | 0.747 | 1.245 |
| rs111577197 | Genotypes | TT | 15 (5.08%) | 14 (4.78%) | 1.10 (0.52-2.35) | 0.805 | 1.006 | 15 (5.64%) | 11 (4.20%) | 1.38 (0.62-3.10) | 0.433 | 2.165 |
|  |  | TC | 100 (33.90%) | 94 (32.08%) | 1.09 (0.77-1.54) | 0.63 | 1.050 | 88 (33.08%) | 86 (32.82%) | 1.03 (0.71-1.49) | 0.878 | 0.878 |
|  |  | CC | 180 (61.02%) | 185 (63.14%) | 1 |  |  | 163 (61.28%) | 165 (62.98%) | 1 |  |  |
|  | Dominant | TT+TC | 115 (38.98%) | 108 (36.86%) | 1.09 (0.78-1.52) | 0.609 | 3.045 | 103 (38.72%) | 97 (37.02%) | 1.07 (0.75-1.52) | 0.709 | 0.886 |
|  |  | CC | 180 (61.02%) | 185 (63.14%) | 1 |  |  | 163 (61.28%) | 165 (62.98%) | 1 |  |  |
|  | Recessive | TT | 15 (5.08%) | 14 (4.78%) | 1.07 (0.51-2.26) | 0.863 | 0.863 | 15 (5.64%) | 11 (4.20%) | 1.37 (0.62-3.04) | 0.441 | 1.103 |
|  |  | TC+CC | 280 (94.92%) | 279 (95.22%) | 1 |  |  | 251 (94.36%) | 251 (95.80%) | 1 |  |  |
|  | Additive | - | - | - | 1.07 (0.81-1.41) | 0.626 | 1.565 | - | - | 1.09 (0.82-1.46) | 0.553 | 0.922 |
| rs3931698 | Genotypes | GG | 2 (0.68%) | 6 (2.05%) | 0.33 (0.07-1.67) | 0.183 | 0.458 | 3 (1.13%) | 10 (3.82%) | 0.26 (0.07-0.97) | **0.045** | 0.113 |
|  |  | GT | 77 (26.10%) | 73 (24.91%) | 1.04 (0.72-1.51) | 0.821 | 1.026 | 64 (24.06%) | 75 (28.63%) | 0.76 (0.51-1.12) | 0.159 | 0.159 |
|  |  | TT | 216 (73.22%) | 214 (73.04%) | 1 |  |  | 199 (74.81%) | 177 (67.56%) | 1 |  |  |
|  | Dominant | GG+GT | 79 (26.78%) | 79 (26.96%) | 0.99 (0.69-1.43) | 0.958 | 0.958 | 67 (25.19%) | 85 (32.44%) | 0.71 (0.48-1.02) | 0.063 | 0.079 |
|  |  | TT | 216 (73.22%) | 214 (73.04%) | 1 |  |  | 199 (74.81%) | 177 (67.56%) | 1 |  |  |
|  | Recessive | GG | 2 (0.68%) | 6 (2.05%) | 0.33 (0.07-1.65) | 0.178 | 0.890 | 3 (1.13%) | 10 (3.82%) | 0.28 (0.77-1.04) | 0.058 | 0.097 |
|  |  | GT+TT | 293 (99.32%) | 287 (97.95%) | 1 |  |  | 263 (98.87%) | 252 (96.18%) | 1 |  |  |
|  | Additive | - | - | - | 0.94 (0.67-1.31) | 0.696 | 1.160 | - | - | 0.68 (0.48-0.95) | **0.025** | 0.125 |
| rs6499221 | Genotypes | AA | 9 (3.05%) | 12 (4.10%) | 0.70 (0.29-1.71) | 0.434 | 0.723 | 9 (3.38%) | 11 (4.20%) | 0.89 (0.36-2.19) | 0.792 | 0.792 |
|  |  | AG | 98 (33.22%) | 104 (35.49%) | 0.89 (0.63-1.26) | 0.520 | 0.520 | 84 (31.58%) | 62 (23.66%) | 1.48 (1.01-2.19) | **0.046** | 0.230 |
|  |  | GG | 188 (63.73%) | 177 (60.41%) | 1 |  |  | 173 (65.04%) | 189 (72.14%) | 1 |  |  |
|  | Dominant | AA+AG | 107 (36.27%) | 116 (39.59%) | 0.87 (0.63-1.22) | 0.424 | 1.060 | 93 (34.96%) | 73 (27.86%) | 1.39 (0.96-2.02) | 0.079 | 0.198 |
|  |  | GG | 188 (63.73%) | 177 (60.41%) | 1 |  |  | 173 (65.04%) | 189 (72.14%) | 1 |  |  |
|  | Recessive | AA | 9 (3.05%) | 12 (4.10%) | 0.74 (0.30-1.76) | 0.482 | 0.603 | 9 (3.38%) | 11 (4.20%) | 0.79 (0.32-1.95) | 0.610 | 0.763 |
|  |  | AG+GG | 286 (96.95%) | 281 (95.90%) | 1 |  |  | 257 (96.62%) | 251 (95.80%) | 1 |  |  |
|  | Additive | - | - | - | 0.87 (0.65-1.17) | 0.355 | 1.775 | - | - | 1.23 (0.90-1.68) | 0.192 | 0.320 |

Abbreviations: SNPs: single-nucleotide polymorphisms; OR: odd ratio; 95% CI: 95% confidence interval; FDR: false discovery rate.

Note: OR and 95% CI were computed by logistic regression analysis with adjustments for age.

^a^ *p* - value was calculated by two -sided χ2 test after adjusted by age with logistic regression analysis.

Bold means statistical significance.

**Table S3.** SNPs rs3931698 (T>G), rs6499221 (G>A) and rs3852740 (C>G) of *LOC105371267* were positively correlated with the PR status, TNM stage and ER status of breast cancer.

|  | **rs3931698 T>G** | | | | |  | **rs6499221 G>A** | | | | |  | **rs3852740 C>G** | | | | |
| --- | --- | --- | --- | --- | --- | --- | --- | --- | --- | --- | --- | --- | --- | --- | --- | --- | --- |
|  |  |  |  |  |  |  |  |  |  |  |  |  |  |  |  |  |  |
| **Characteristics** | **Homozygote** | **Heterozygote** | **Dominant** | **Recessive** | **Additive** | **Characteristics** | **Homozygote** | **Heterozygote** | **Dominant** | **Recessive** | **Additive** | **Characteristics** | **Homozygote** | **Heterozygote** | **Dominant** | **Recessive** | **Additive** |
|  | **(GG vs TT)** | **(GT vs TT)** | **(GG +GT vs TT)** | **(GG vs GT + TT)** | **(GG vs GT vs TT)** |  | **(AA vs GG)** | **(AG vs GG)** | **(AA+AG vs GG)** | **(AA vs AG + GG)** | **(AA vs AG vs GG)** |  | **(GG vs CC)** | **(GC vs CC)** | **(GG + GC vs CC)** | **(GG vs GC + CC)** | **(GG vs GC vs CC)** |
| PR status [Positive (n = 328) vs negative (n = 224)] | | | | | | ER status [Positive (n = 380) vs negative (n = 172)] | | | | | | ER status [Positive (n = 380) vs negative (n = 172)] | | | | | |
| OR (95% CI) | 1.11 (0.18-6.72) | 1.52 (1.01-2.29) | 1.50 (1.01-2.25) | 1.00 (0.17-6.06) | 1.44 (0.99-2.11) | OR (95% CI) | 4.21 (0.95-18.66) | 1.27 (0.86-1.88) | 1.38 (0.94-2.03) | 3.90 (0.88-17.19) | 1.43 (1.02-2.02) | OR (95% CI) | 0.52 (0.22-1.22) | 0.73 (0.49-1.08) | 0.70 (0.48-1.02) | 0.58 (0.25-1.34) | 0.73 (0.53-0.99) |
| *p*-value ^a^ | 0.912 | **0.043** | **0.046** | 1 | 0.059 | *p*-value ^a^ | 0.058 | 0.232 | 0.1 | 0.072 | **0.041** | *p*-value ^a^ | 0.133 | 0.113 | 0.06 | 0.201 | **0.043** |
| FDR test | 1.14 | 0.215 | 0.115 | 1 | 0.098 | FDR test | 0.145 | 0.232 | 0.125 | 0.12 | 0.205 | FDR test | 0.166 | 0.188 | 0.15 | 0.201 | 0.215 |
| TNM stage [(III-IV (n = 161) vs I-II (n = 366)] | | | | | |  |  |  |  |  |  |  |  |  |  |  |  |
| OR (95% CI) | / | 1.58 (1.04-2.40) | 1.48 (0.98-2.25) | / | 1.33 (0.90-1.97) |  |  |  |  |  |  |  |  |  |  |  |  |
| *p*-value ^a^ | / | **0.033** | 0.063 | / | 0.151 |  |  |  |  |  |  |  |  |  |  |  |  |
| FDR test | / | 0.099 | 0.095 | / | 0.151 |  |  |  |  |  |  |  |  |  |  |  |  |

Abbreviations: SNPs: single-nucleotide polymorphisms; OR: odd ratio; 95% CI: 95% confidence interval; FDR: false discovery rate. PR: progesterone receptor, ER: estrogen receptor, TNM: primary tumor, regional lymph nodes, and distant metastasis

Note: OR and 95% CI were computed by logistic regression analysis with adjustments for age.

^a^ *p* - value was calculated by two -sided χ2 test after adjusted by age with logistic regression analysis.

Bold means statistical significance.

**Table S4.** Relationships of *LOC105371267* rs3931698 T>G and clinical characteristics of breast cancer risk.

| **Characteristics** | **Homozygote**  **(GG vs TT)** | **Heterozygote**  **(GT vs TT)** | **Dominant**  **(GG +GT vs TT)** | **Recessive**  **(GG vs GT + TT)** | **Additive**  **(GG vs GT vs TT)** |
| --- | --- | --- | --- | --- | --- |
| ER status [Positive (n = 380) vs negative (n = 172)] | | | | | |
| OR (95% CI) | 0.72 (0.12-4.39) | 1.43 (0.92-2.22) | 1.39 (0.91-2.14) | 0.67 (0.11-4.03) | 1.32 (0.88-1.98) |
| *p*-value ^a^ | 0.726 | 0.109 | 0.131 | 0.658 | 0.181 |
| FDR test | 0.726 | 0.545 | 0.328 | 0.823 | 0.302 |
| Ki67 status [High (n = 371) vs low (n = 154)] | | | | | |
| OR (95% CI) | / | 0.78 (0.51-1.20) | 0.82 (0.54-1.25) | / | 0.88 (0.59-1.32) |
| *p*-value ^a^ | / | 0.261 | 0.359 | / | 0.533 |
| FDR test | / | 0.783 | 0.539 | / |  |
| Tumor size [> 2 cm (n = 238) vs ≤ 2 cm (n = 206)] | | | | | |
| OR (95% CI) | 0.87 (0.02-6.28) | 1.00 (0.64-1.55) | 0.99 (0.64-1.53) | 0.87 (0.12-6.27) | 0.99 (0.65-1.49) |
| *p*-value ^a^ | 0.893 | 0.985 | 0.967 | 0.894 | 0.947 |
| FDR test | 4.465 | 0.985 | 1.209 | 2.235 | 1.578 |
| Lymph nodes metastasis [Positive (n = 277) vs negative (n = 279)] | | | | | |
| OR (95% CI) | 4.27 (0.47-38.53) | 1.22 (0.83-1.80) | 1.27 (0.87-1.86) | 4.06 (0.45-36.57) | 1.30 (0.91-1.87) |
| *p*-value ^a^ | 0.196 | 0.308 | 0.221 | 0.211 | 0.152 |
| FDR test | 0.490 | 0.308 | 0.276 | 0.352 | 0.760 |

Abbreviations: OR: odd ratio; 95% CI: 95% confidence interval. ER: estrogen receptor, TNM: primary tumor, regional lymph nodes, and distant metastasis

Note: OR and 95% CI were computed by logistic regression analysis with adjustments for age.

^a^ *p* - value was calculated by two -sided χ2 test after adjusted by age with logistic regression analysis.

“/” denotes that the values of OR (95% CI) and *p* were unavailable due to the absence of allele or genotype frequency.

**Table S5.** Relationships of *LOC105371267* rs6499221 G>A and clinical characteristics of breast cancer.

| **Characteristics** | **Homozygote**  **(AA vs GG)** | **Heterozygote**  **(AG vs GG)** | **Dominant**  **(AA+AG vs GG)** | **Recessive**  **(AA vs AG + GG)** | **Additive**  **(AA vs AG vs GG)** |
| --- | --- | --- | --- | --- | --- |
| PR status [Positive (n = 328) vs negative (n = 224)] | | | | | |
| OR (95% CI) | 2.03 (0.70-5.85) | 1.15 (0.80-1.67) | 1.21 (0.85-1.73) | 1.94 (0.68-5.54) | 1.23 (0.90-1.69) |
| *p*-value ^a^ | 0.131 | 0.453 | 0.298 | 0.218 | 0.191 |
| FDR test | 0.655 | 0.453 | 0.373 | 0.363 | 0.478 |
| Ki67 status [High (n = 371) vs low (n = 154)] | | | | | |
| OR (95% CI) | 0.50 (0.19-1.31) | 0.80 (0.53-1.20) | 0.76 (0.51-1.12) | 0.54 (0.21-1.40) | 0.76 (0.55-1.06) |
| *p*-value ^a^ | 0.157 | 0.273 | 0.165 | 0.203 | 0.105 |
| FDR test | 0.683 | 0.525 | 0.458 | 0.491 | 1.269 |
| Tumor size [> 2 cm (n = 238) vs ≤ 2 cm (n = 206)] | | | | | |
| OR (95% CI) | 1.93 (0.64-5.81) | 1.17 (0.79-1.76) | 1.23 (0.83-1.81) | 1.83 (0.61-5.47) | 1.24 (0.88-1.75) |
| *p*-value ^a^ | 0.243 | 0.436 | 0.303 | 0.279 | 0.212 |
| FDR test | 0.608 | 0.436 | 0.379 | 0.465 | 1.060 |
| Lymph nodes metastasis [Positive (n = 277) vs negative (n = 279)] | | | | | |
| OR (95% CI) | 0.52 (0.19-1.42) | 1.15 (0.81-1.65) | 1.08 (0.76-1.52) | 0.50 (0.18-1.34) | 0.99 (0.73-1.33) |
| *p*-value ^a^ | 0.201 | 0.433 | 0.680 | 0.168 | 0.926 |
| FDR test | 0.503 | 0.722 | 0.850 | 0.840 | 0.926 |
| TNM stage [(III-IV (n = 161) vs I-II (n = 366)] | | | | | |
| OR (95% CI) | 0.96 (0.33-2.76) | 1.20 (0.81-1.79) | 1.18 (0.80-1.73) | 0.90 (0.31-2.57) | 1.12 (0.80-1.56) |
| *p*-value ^a^ | 0.935 | 0.360 | 0.398 | 0.839 | 0.504 |
| FDR test | 0.935 | 1.800 | 0.995 | 1.049 | 0.840 |

Abbreviations: OR: odd ratio; 95% CI: 95% confidence interval. PR: progesterone receptor, TNM: primary tumor, regional lymph nodes, and distant metastasis

Note: OR and 95% CI were computed by logistic regression analysis with adjustments for age.

^a^ *p* - value was calculated by two -sided χ2 test after adjusted by age with logistic regression analysis.

**Table S6.** Relationships of *LOC105371267* rs8044565 T>C and clinical characteristics of breast cancer.

| **Characteristics** | **Homozygote**  **(CC vs TT)** | **Heterozygote**  **(CT vs TT)** | **Dominant**  **(CC +CT vs TT)** | **Recessive**  **(CC vs CT + TT)** | **Additive**  **(CC vs CT vs TT)** |
| --- | --- | --- | --- | --- | --- |
| ER status [Positive (n = 380) vs negative (n = 172)] | | | | | |
| OR (95% CI) | 0.60 (0.30-1.19) | 1.01 (0.69-1.49) | 0.92 (0.64-1.33) | 0.60 (0.31-1.17) | 0.87 (0.65-1.16) |
| *p*-value ^a^ | 0.144 | 0.959 | 0.664 | 0.132 | 0.336 |
| FDR test | 0.360 | 0.959 | 0.830 | 0.660 | 0.560 |
| PR status [Positive (n = 328) vs negative (n = 224)] | | | | | |
| OR (95% CI) | 0.54 (0.27-1.07) | 1.10 (0.76-1.58) | 0.97 (0.69-1.37) | 0.53 (0.27-1.02) | 0.88 (0.67-1.16) |
| *p*-value ^a^ | 0.077 | 0.628 | 0.875 | 0.056 | 0.362 |
| FDR test | 0.193 | 0.785 | 0.875 | 0.280 | 0.603 |
| Ki67 status [High (n = 371) vs low (n = 154)] | | | | | |
| OR (95% CI) | 1.15 (0.52-2.45) | 0.78 (0.53-1.17) | 0.83 (0.57-1.22) | 1.26 (0.58-2.75) | 0.923 (0.681.25) |
| *p*-value ^a^ | 0.739 | 0.739 | 0.343 | 0.565 | 0.604 |
| FDR test | 0.739 | 0.924 | 1.715 | 1.715 | 1.007 |
| Tumor size [> 2 cm (n = 238) vs ≤ 2 cm (n = 206)] | | | | | |
| OR (95% CI) | 2.05 (093-4.53) | 1.22 (0.82-1.82) | 0.32 (0.91-1.93) | 0.90 (0.87-1.13) | 1.33 (0.98-1.80) |
| *p*-value ^a^ | 0.076 | 0.323 | 0.146 | 0.107 | 0.069 |
| FDR test | 0.190 | 0.323 | 0.183 | 0.178 | 0.345 |
| Lymph nodes metastasis [Positive (n = 277) vs negative (n = 279)] | | | | | |
| OR (95% CI) | 0.77 (0.39-1.52) | 1.05 (0.74-1.49) | 1.00 (0.71-1.39) | 0.75 (0.38-1.85) | 0.95 (0.73-1.25) |
| *p-*value ^a^ | 0.445 | 0.805 | 0.980 | 0.407 | 0.723 |
| FDR test | 1.113 | 1.006 | 0.980 | 2.035 | 1.205 |
| TNM stage [(III-IV (n = 161) vs I-II (n = 366)] | | | | | |
| OR (95% CI) | 0.66 (0.29-1.51) | 1.26 (0.86-1.87) | 1.15 (0.79-1.67) | 0.61 (0.27-1.34) | 1.33 (0.98-1.81) |
| *p*-value ^a^ | 0.328 | 0.240 | 0.464 | 0.222 | 0.068 |
| FDR test | 0.410 | 0.400 | 0.464 | 0.555 | 0.340 |

Abbreviations: OR: odd ratio; 95% CI: 95% confidence interval. PR: progesterone receptor, ER: estrogen receptor, TNM: primary tumor, regional lymph nodes, and distant metastasis

Note: OR and 95% CI were computed by logistic regression analysis with adjustments for age.

^a^ *p* - value was calculated by two -sided χ2 test after adjusted by age with logistic regression analysis.

**Table S7.** Relationships of *LOC105371267* rs3852740 C>G and clinical characteristics of breast cancer.

| **Characteristics** | **Homozygote**  **(GG vs CC)** | **Heterozygote**  **(GC vs CC)** | **Dominant**  **(GG + GC vs CC)** | **Recessive**  **(GG vs GC + CC)** | **Additive**  **(GG vs GC vs CC)** |
| --- | --- | --- | --- | --- | --- |
| PR status [Positive (n = 328) vs negative (n = 224)] | | | | | |
| OR (95% CI) | 0.70 (0.30-1.63) | 0.84 (0.58-1.21) | 0.82 (0.58-1.17) | 0.74 (0.32-1.72) | 0.84 (0.62-1.13) |
| *p*-value ^a^ | 0.407 | 0.345 | 0.270 | 0.483 | 0.240 |
| FDR test | 0.509 | 0.575 | 0.675 | 0.483 | 1.2 |
| Ki67 status [High (n = 371) vs low (n = 154)] | | | | | |
| OR (95% CI) | 2.43 (0.69-8.52) | 0.97 (0.64-1.45) | 1.05 (0.71-1.56) | 2.46 (0.71-8.55) | 1.13 (0.80-1.59) |
| *p*-value ^a^ | 0.166 | 0.866 | 0.819 | 1.411 | 0.489 |
| FDR test | 0.830 | 1.083 | 1.365 | 1.411 | 1.223 |
| Tumor size [> 2 cm (n = 238) vs ≤ 2 cm (n = 206)] | | | | | |
| OR (95% CI) | 1.36 (0.51-3.62) | 1.01 (0.67-1.51) | 1.04 (0.71-1.54) | 1.36 (0.52-3.58) | 1.07 (0.77-1.49) |
| *p*-value ^a^ | 0.534 | 0.975 | 0.841 | 0.533 | 0.698 |
| FDR test | 1.335 | 0.975 | 1.051 | 2.665 | 1.163 |
| Lymph nodes metastasis [Positive (n = 277) vs negative (n = 279)] | | | | | |
| OR (95% CI) | 1.07 (0.46-2.48) | 0.90 (0.62-1.29) | 0.92 (0.65-1.30) | 1.11 (0.48-2.55) | 0.95 (0.71-1.28) |
| *p*-value ^a^ | 0.879 | 0.571 | 0.633 | 0.815 | 0.748 |
| FDR test | 0.879 | 2.855 | 1.583 | 1.019 | 1.247 |
| TNM stage [(III-IV (n = 161) vs I-II (n = 366)] | | | | | |
| OR (95% CI) | 1.01 (0.39-2.57) | 0.71 (0.47-1.07) | 0.74 (0. 50-1.09) | 1.13 (0.45-2.85) | 0.81 (0.58-1.14) |
| *p*-value ^a^ | 0.990 | 0.099 | 0.128 | 0.804 | 0.229 |
| FDR test | 0.990 | 0.495 | 0.320 | 1.005 | 0.382 |

Abbreviations: OR: odd ratio; 95% CI: 95% confidence interval. PR: progesterone receptor, TNM: primary tumor, regional lymph nodes, and distant metastasis

Note: OR and 95% CI were computed by logistic regression analysis with adjustments for age.

^a^ *p* - value was calculated by two -sided χ2 test after adjusted by age with logistic regression analysis.

**Table S8.** Relationships of *LOC105371267* rs111577197 and clinical characteristics of breast cancer.

| **Characteristics** | **Homozygote**  **(TT vs CC)** | **Heterozygote**  **(TC vs CC)** | **Dominant**  **(TT + TC vs CC)** | **Recessive**  **(TT vs TC + CC)** | **Additive**  **(TT vs TC vs CC)** |
| --- | --- | --- | --- | --- | --- |
| ER status [Positive (n = 380) vs negative (n = 172)] | | | | | |
| OR (95% CI) | 2.38 (0.89-6.40) | 1.04 (0.71-1.54) | 1.15 (0.79-1.67) | 2.34 (0.88-6.24) | 1.22 (0.90-1.67) |
| *p*-value ^a^ | 0.085 | 0.826 | 0.464 | 0.088 | 0.207 |
| FDR test | 0.425 | 0.826 | 0.580 | 0.220 | 0.345 |
| PR status [Positive (n = 328) vs negative (n = 224)] | | | | | |
| OR (95% CI) | 2.26 (0.94-5.44) | 0.91 (0.63-1.31) | 1.02 (0.72-1.45) | 2.34 (0.98-5.57) | 1.13 (0.85-1.51) |
| *p*-value ^a^ | 0.069 | 0.618 | 0.916 | 0.055 | 0.405 |
| FDR test | 0.173 | 0.773 | 0.916 | 0.275 | 0.675 |
| Ki67 status [High (n = 371) vs low (n = 154)] | | | | | |
| OR (95% CI) | 0.61 (0.26-1.45) | 0.77 (0.52-1.15) | 0.75 (0.51-1.10) | 0.67 (0.28-1.58) | 0.78 (0.56-1.07) |
| *p*-value ^a^ | 0.264 | 0.208 | 0.144 | 0.358 | 0.120 |
| FDR test | 0.330 | 0.347 | 0.360 | 0.358 | 0.600 |
| Tumor size [> 2 cm (n = 238) vs ≤ 2 cm (n = 206)] | | | | | |
| OR (95% CI) | 1.34 (0.52-3.03) | 0.94 (0.63-1.41) | 0.99 (0.67-1.45) | 1.37 (0.58-3.24) | 1.04 (0.76-1.42) |
| *p*-value ^a^ | 0.508 | 0.767 | 0.946 | 0.472 | 0.830 |
| FDR test | 1.270 | 1.278 | 0.946 | 2.360 | 1.038 |
| Lymph nodes metastasis [Positive (n = 277) vs negative (n = 279)] | | | | | |
| OR (95% CI) | 1.86 (0.86-4.04) | 0.12 (0.78-1.60) | 0.20 (0.85-1.69) | 1.79 (0.84-3.84) | 0.23 (0.93-1.62) |
| *p*-value ^a^ | 0.114 | 0.540 | 0.299 | 0.134 | 0.155 |
| FDR test | 0.570 | 0.540 | 0.374 | 0.335 | 0.258 |
| TNM stage [(III-IV (n = 161) vs I-II (n = 366)] | | | | | |
| OR (95% CI) | 1.70 (0.76-3.77) | 1.36 (0.93-2.01) | 1.40 (0.96-2.04) | 1.52 (0.69-3.32) | 1.33 (0.98-1.81) |
| *p*-value ^a^ | 0.194 | 0.131 | 0.081 | 0.298 | 0.068 |
| FDR test | 0.243 | 0.218 | 0.405 | 0.298 | 0.170 |

Abbreviations: OR: odd ratio; 95% CI: 95% confidence interval. PR: progesterone receptor, ER: estrogen receptor, TNM: primary tumor, regional lymph nodes, and distant metastasis.

Note: OR and 95% CI were computed by logistic regression analysis with adjustments for age.

^a^ *p* - value was calculated by two -sided χ2 test after adjusted by age with logistic regression analysis.

**Table S9.** Primers used in this study.

| SNP | **1^st^-PCRP** | **2^nd^-PCRP** | **UEP-DIR** | **UEP-SEQ** |
| --- | --- | --- | --- | --- |
| rs6499221 | ACGTTGGATGTTTGAGCTCTAAAGACCAGG | ACGTTGGATGACATTCAAAGGTCCCAGGTG | F | gGGGAGGAGACTATTCAATGC |
| rs3931698 | ACGTTGGATGTTGATGAAGCCAACGTGAGG | ACGTTGGATGACTGTAACCTGGGCTGAATG | F | cccgtCAAGTTGAAAGTGTTTCCCG |
| rs8044565 | ACGTTGGATGGCGCCCATCAAGATAAACTC | ACGTTGGATGATTGCACCATGACCATGCAC | R | gaggGTGAGACTCCGTACCAAAAA |
| rs3852740 | ACGTTGGATGTGTGGCCATAGAAACGGAAC | ACGTTGGATGGCCAGCGTTAAATAACCATC | F | CGTTAAATAACCATCCTCTTG |
| rs111577197 | ACGTTGGATGAGTGGAGCGATCATGCATGG | ACGTTGGATGTAGTTCTAGCCACTGAGGAG | R | GCTTGAGCCCAGAAG |

Abbreviations: SNP: single-nucleotide polymorphism; PCRP: PCR primer; UEP: unique base extension prime

**Table S10.** Results of association analysis of haplotype of *LOC105371267* and the risk of breast cancer.

| SNP | Haplotype | Frequency | *χ*^2^ | *p*-value | FDR test |
| --- | --- | --- | --- | --- | --- |
|  |  | (case/control) |  |  |  |
| rs8044565\|rs3931698 | TT | 0.62/0.59 | 1.66 | 0.198 | 0.495 |
|  | TC | 0.25/0.24 | 0.00 | 0.958 | 1.597 |
|  | GT | 0.13/0.16 | 3.36 | 0.067 | 0.335 |

Abbreviations: OR: odds ratio; CI: confidence interval.

Note: ^a^ *p* - value was calculated by two -sided χ2 test after adjusted by age with logistic regression analysis.

**Table S11.** The genotype combination of SNP-SNP interaction among variants of *LOC105371267* in breast cancer risk analysis using MDR method .

| Combination | class '1' | class '0' | 1/0 Ratio | Predicted 'class' |
| --- | --- | --- | --- | --- |
| rs111577197,rs3852740,rs3931698,rs6499221,rs8044565 |  |  |  |  |
| TT,CC,TT,AG,TC | 7.0 | 8.0 | 0.875 | 0 |
| TT,CC,TT,AG,CC | 1.0 | 0.0 | ∞ | 1 |
| TT,CC,TT,AG,TT | 1.0 | 0.0 | ∞ | 1 |
| TT,CC,TT,GG,TT | 11.0 | 10.0 | 1.1 | 1 |
| TT,CC,TT,AA,CC | 0.0 | 1.0 | 0.0 | 0 |
| TT,CC,GG,GG,TT | 0.0 | 1.0 | 0.0 | 0 |
| TT,CC,GT,AG,TC | 4.0 | 1.0 | 4.0 | 1 |
| TT,CC,GT,AG,TT | 0.0 | 2.0 | 0.0 | 0 |
| TT,CC,GT,GG,TT | 3.0 | 2.0 | 1.5 | 1 |
| TT,CG,TT,GG,TT | 2.0 | 0.0 | ∞ | 1 |
| CC,CC,TT,AG,TC | 42.0 | 34.0 | 1.2353 | 1 |
| CC,CC,TT,AG,CC | 7.0 | 4.0 | 1.75 | 1 |
| CC,CC,TT,AG,TT | 1.0 | 0.0 | ∞ | 1 |
| CC,CC,TT,GG,TC | 12.0 | 16.0 | 0.75 | 0 |
| CC,CC,TT,GG,CC | 3.0 | 1.0 | 3.0 | 1 |
| CC,CC,TT,GG,TT | 70.0 | 63.0 | 1.1111 | 1 |
| CC,CC,TT,AA,TC | 0.0 | 1.0 | 0.0 | 0 |
| CC,CC,TT,AA,CC | 7.0 | 6.0 | 1.1667 | 1 |
| CC,CC,GG,AG,TT | 0.0 | 1.0 | 0.0 | 0 |
| CC,CC,GG,GG,TT | 1.0 | 9.0 | 0.1111 | 0 |
| CC,CC,GT,AG,TC | 10.0 | 13.0 | 0.7692 | 0 |
| CC,CC,GT,AG,TT | 2.0 | 1.0 | 2.0 | 1 |
| CC,CC,GT,GG,TC | 7.0 | 8.0 | 0.875 | 0 |
| CC,CC,GT,GG,TT | 42.0 | 41.0 | 1.0244 | 1 |
| CC,GG,TT,AG,TC | 1.0 | 6.0 | 0.1667 | 0 |
| CC,GG,TT,GG,TC | 0.0 | 2.0 | 0.0 | 0 |
| CC,GG,TT,GG,TT | 16.0 | 12.0 | 1.3333 | 1 |
| CC,GG,GT,GG,TT | 3.0 | 0.0 | ∞ | 1 |
| CC,CG,TT,AG,TC | 21.0 | 22.0 | 0.9545 | 0 |
| CC,CG,TT,AG,CC | 6.0 | 2.0 | 3.0 | 1 |
| CC,CG,TT,GG,TC | 10.0 | 13.0 | 0.7692 | 0 |
| CC,CG,TT,GG,CC | 0.0 | 1.0 | 0.0 | 0 |
| CC,CG,TT,GG,TT | 53.0 | 59.0 | 0.8983 | 0 |
| CC,CG,TT,AA,TC | 1.0 | 0.0 | ∞ | 1 |
| CC,CG,TT,AA,CC | 2.0 | 6.0 | 0.3333 | 0 |
| CC,CG,GG,GG,TT | 1.0 | 0.0 | ∞ | 1 |
| CC,CG,GT,AG,TC | 2.0 | 6.0 | 0.3333 | 0 |
| CC,CG,GT,AG,TT | 0.0 | 2.0 | 0.0 | 0 |
| CC,CG,GT,GG,TC | 1.0 | 1.0 | 1.0 | 1 |
| CC,CG,GT,GG,TT | 18.0 | 20.0 | 0.9 | 0 |
| CC,CG,GT,AA,TC | 0.0 | 1.0 | 0.0 | 0 |
| TC,CC,TT,AG,TC | 42.0 | 31.0 | 1.3548 | 1 |
| TC,CC,TT,AG,CC | 4.0 | 5.0 | 0.8 | 0 |
| TC,CC,TT,AG,TT | 1.0 | 1.0 | 1.0 | 1 |
| TC,CC,TT,GG,TC | 8.0 | 8.0 | 1.0 | 1 |
| TC,CC,TT,GG,CC | 1.0 | 0.0 | ∞ | 1 |
| TC,CC,TT,GG,TT | 28.0 | 35.0 | 0.8 | 0 |
| TC,CC,TT,AA,TC | 0.0 | 2.0 | 0.0 | 0 |
| TC,CC,TT,AA,CC | 5.0 | 4.0 | 1.25 | 1 |
| TC,CC,GG,GG,TT | 3.0 | 5.0 | 0.6 | 0 |
| TC,CC,GT,AG,TC | 5.0 | 13.0 | 0.3846 | 0 |
| TC,CC,GT,AG,TT | 2.0 | 0.0 | ∞ | 1 |
| TC,CC,GT,GG,TC | 1.0 | 3.0 | 0.3333 | 0 |
| TC,CC,GT,GG,TT | 28.0 | 24.0 | 1.1667 | 1 |
| TC,GG,TT,GG,TT | 3.0 | 0.0 | ∞ | 1 |
| TC,CG,TT,AG,TC | 18.0 | 11.0 | 1.6364 | 1 |
| TC,CG,TT,AG,CC | 0.0 | 1.0 | 0.0 | 0 |
| TC,CG,TT,GG,TC | 2.0 | 3.0 | 0.6667 | 0 |
| TC,CG,TT,GG,TT | 22.0 | 22.0 | 1.0 | 1 |
| TC,CG,TT,AA,CC | 2.0 | 2.0 | 1.0 | 1 |
| TC,CG,GT,AG,TC | 3.0 | 2.0 | 1.5 | 1 |

Abbreviation: MDR, Multifactor Dimensionality Reduction.

Note: Yellow backgroud implies the best model which with the highest 1/0 Ratio value.
