# Supplementary material for: Impacts of LOC105371267 Variants on Breast Cancer Susceptibility in Northern Chinese Han Females: A Population-Based Case-Control Study
Source: J Oncol. 2021 Aug 28;2021:4990695. doi: 10.1155/2021/4990695 (PMC8407995; doi:10.1155/2021/4990695)
Supplement: Supplementary Materials — Figure S1: flow diagram of study design. Table S1: association analysis results of genetic polymorphisms of LOC105371267 and breast cancer susceptibility. Table S2: results of stratification analysis between LOC105371267 polymorphisms and breast cancer risk by age of 52 years. Table S3: SNPs rs3931698 (T > G), rs6499221 (G > A), and rs3852740 (C > G) of LOC105371267 were positively correlated with the PR status, TNM stage, and ER status of breast cancer. Table S4: relationships of LOC105371267 rs3931698 (T > G) and clinical characteristics of breast cancer risk. Table S5: relationships of LOC105371267 rs6499221 (G > A) and clinical characteristics of breast cancer. Table S6: relationships of LOC105371267 rs8044565 (T > C) and clinical characteristics of breast cancer. Table S7: relationships of LOC105371267 rs3852740 (C > G) and clinical characteristics of breast cancer. Table S8: relationships of LOC105371267 rs111577197 and clinical characteristics of breast cancer. Table S9: primers used in this study. Table S10: results of association analysis of haplotype of LOC105371267 and the risk of breast cancer. Table S11: the genotype combination of SNP-SNP interaction among variants of LOC105371267 in breast cancer risk analysis using MDR method. . [file 4990695.f1.zip › 4990695.f1/Figure S1-BC.doc]

Experiment: Association study of genetic polymorphism of *LOC105371267* gene and breast cancer risk

Sample collection of Case and Control groups

561 patients with BC (cases) and 555 healthy individuals (controls)(Shaanxi Provincial Cancer Hospital)

DNA Extraction

SNP Sites Selection

Primer Design

Sequenom MassARRAY SNP genotyping

Data collation and analysis

Examination of Hardy–Weinberg equilibrium (HWE) analysis

Single nuleotide polymorphism(SNP) analysis

Linkage disequilibrium(LD) analysis

Haplotype analysis

Multifactor Dimensionality Reduction (MDR) analysis

False-positive report probability (FPRP) analysis

Association analysis in overall and subgroup
